# Nutritional aspects in neuroendocrine neoplasms. bridging the gap between dietary interventions and cancer care strategies: a scoping review

**DOI:** 10.1007/s40618-024-02462-8

**Published:** 2024-10-12

**Authors:** Sara Massironi, Francesco Panzuto, Alessandra Zilli, Maria Rinzivillo, Ambra Ciliberto, Elena Romano, Silvio Danese, Alessandro Laviano

**Affiliations:** 1 Division of Gastroenterology Fondazione, IRCCS San Gerardo dei Tintori, Monza, Italia; 2 Department of Surgical-Medical Sciences and Translational Medicine, Digestive Disease Unit, Sapienza University of Rome, Sant’Andrea University Hospital, ENETS Center of Excellence, Rome, Italy; 3 Gastroenterology and Endoscopy, IRCCS San Raffaele Hospital, Milan, Italy; 4 Vita-Salute San Raffaele University, Milan, Italy; 5 Department of Translational and Precision Medicine, Clinical Nutrition Unit, Sapienza University of Rome, Sant’Andrea University Hospital, Rome, Italy; 6 Division of Gastroenterology, San Gerardo Hospital, Via Pergolesi 3, Monza, Italy

**Keywords:** Neuroendocrine neoplasms, Nutritional management, Dietary interventions, Micronutrient deficiencies, Personalized cancer care

## Abstract

**Purpose:**

Neuroendocrine neoplasms (NENs) represent heterogeneous tumors arising from neuroendocrine cells in different organs. Despite growing interest in the nutritional aspects of NEN management, research in this area is limited. Aim of this review is to summarize the current state of knowledge, highlight research gaps, and underscore the significance of nutrition in the comprehensive care of NEN patients.

**Methods:**

We conducted an extensive bibliographic search focusing on studies (including retrospective and prospective studies, systematic reviews, case series, and guidelines) exploring the relationship between nutritional assessments, dietary interventions, micronutrient deficiencies, and their impact on NEN outcomes.

**Results:**

Significant gaps exist in current research, particularly in understanding the specific nutritional needs of NEN patients and how tailored nutritional interventions can improve clinical outcomes. Evidence suggests that a high-fat Western diet may promote the growth of NEN, while a Mediterranean diet may help lower insulin levels and strengthen the immune system, potentially preventing tumor development. The ketogenic diet and intermittent fasting may also have positive impacts. Addressing common micronutrient deficiencies, such as vitamin D and niacin, is crucial to mitigate disease progression. There’s a crucial need for future studies to include a comprehensive nutritional assessment incorporating patient-reported outcomes, to fully capture the impact of nutritional strategies.

**Conclusion:**

Nutritional management, an important but under-researched facet of NEN treatment, significantly improves patients’ quality of life and survival. Integrating nutrition into personalized cancer care is essential, highlighting the role of nutritional strategies in optimizing patient outcomes.

**Supplementary Information:**

The online version contains supplementary material available at 10.1007/s40618-024-02462-8.

## Introduction

Neuroendocrine neoplasms (NENs) represent a complex and heterogeneous group of tumors originating from neuroendocrine cells located throughout the body. They are characterized by their unique ability to synthesize and secrete a variety of peptides, amines, and hormones, leading to various clinical syndromes [1]. NENs are classified as well-differentiated neuroendocrine tumors (NETs) and poorly differentiated neuroendocrine carcinomas (NECs). Well-differentiated NETs are further subcategorized based on the Ki-67 proliferation index into low-grade (G1, Ki-67 < 3%), intermediate-grade (G2, Ki-67 3–20%), and high-grade (G3, Ki-67 > 20%). Poorly differentiated NECs are always classified as high-grade (G3) with a Ki-67 index typically exceeding 50% [2, 3]. NENs often remain asymptomatic for long periods, complicating their detection and treatment, making them a significant concern in oncology [4, 5].

The global incidence and prevalence of NENs have been increasing, reflecting advances in diagnostic techniques and growing awareness among the medical community [4, 6–12]. Yet, the underlying causes of NENs remain predominantly elusive, with identified risk factors being a mix of genetic predisposition — such as mutations in the MEN1 (Multiple Endocrine Neoplasia type 1), VHL (Von Hippel-Lindau), TSC2 (Tuberous Sclerosis Complex 2), and NF1 (Neurofibromatosis type 1) genes — and environmental exposures [13]. Despite the etiologic factors underlying NENs remaining largely unexplained, potentially modifiable risk factors have been identified as dietary habits and nutritional status.

Nutrition, a cornerstone of overall health, significantly impacts the development and progression of various diseases, including cancer. While the relationship between dietary patterns, nutrient intake, and cancer risk and prognosis has been extensively studied, the specific impact of diet on the development and progression of NENs and the impact of these tumors on patients’ nutritional well-being should be further investigated.

Emerging research findings suggest that nutritional factors could influence NEN development. For instance, a high-fat diet, typical of Western eating habits, is associated with an increased risk of developing cancers [14], including NENs [15]. Conversely, a Mediterranean diet (MD), rich in fruits, vegetables, whole grains, and lean proteins, may offer protective effects against these tumors [15, 16]. Such a diet, rich in antioxidants and anti-inflammatory nutrients, may indeed modulate various metabolic and immune pathways, potentially influencing tumor growth and patient outcomes.

On the other hand, depending on their location and biological behavior, NENs can significantly affect a patient’s nutritional status. In particular, gastrointestinal NEN may lead to malabsorption syndromes, nutritional deficiencies, and unintentional weight loss, further complicating the management of the disease and affecting the quality of life [17, 18]. Indeed, metabolic and nutritional complications, including malnutrition, sarcopenia, and obesity, can further complicate the clinical picture. Malnutrition is a critical issue in NEN patients, often leading to a decline in functional status, increased treatment-related complications, and poorer overall prognosis [17, 18]. Malnutrition exacerbates sarcopenia, characterized by the loss of skeletal muscle mass and function, creating a vicious cycle that impairs physical function and reduces survival rates [18]. Sarcopenia is particularly prevalent among NEN patients and can significantly impact their prognosis and quality of life [19, 20]. Conversely, obesity, especially visceral adipose tissue accumulation, is linked to increased inflammation and can negatively affect treatment outcomes. Sarcopenic obesity, where obesity coexists with sarcopenia, adds another layer of complexity, influencing treatment responses and overall outcomes. Indeed, chronic inflammation, driven both by poor nutrition and excess visceral fat, is associated with worse clinical outcomes in NEN patients. This inflammation can influence the tumor microenvironment, potentially promoting tumor growth and resistance to therapy [21].

This review aims to comprehensively examine the current literature on the prevalence, causes, and clinical impact of malnutrition, sarcopenia, and obesity in NENs patients, exploring the intersection of nutrition and NENs. By identifying existing knowledge gaps, this review seeks to propose future research directions to enhance the nutritional management of NEN patients and improve clinical outcomes. We specifically explore a range of nutritional interventions—such as dietary patterns, supplements, and strategies to address both undernutrition, overnutrition, and sarcopenia—to underscore the critical link between nutrition and NEN.

## Methods

A systematic search strategy was employed to identify and map the available evidence on the role of nutrition in NENs. We focused our search on studies published between January 2003 and December 2023, exploring the relationship between nutritional assessments, dietary interventions, micronutrient deficiencies, and their impact on NEN outcomes. The primary databases used for this search were PubMed, Scopus, and Google Scholar, to capture the broadest possible range of both indexed and grey literature. Table 1 provides a PRISMA flow diagram of the study selection process.

**Table 1:**
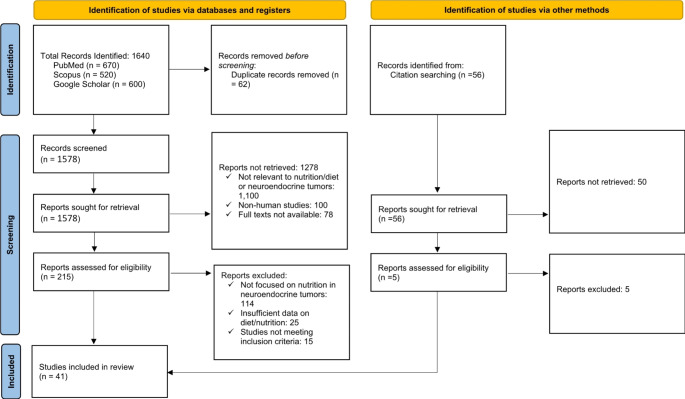
provides a PRISMA flow diagram for a clear visualization of the study selection process for our review

*Keywords and Search Terms*.

The search was structured using combinations of specific keywords and terms from the Medical Subject Headings (MeSH) to maximize the retrieval of relevant studies. Primary keywords included “Neuroendocrine Neoplasms”, “Neuroendocrine Tumors”, “NEN”, “NET”, “Carcinoid Tumors”.

and “Nutrition”, “Diet”, “Dietary Patterns”, “Nutritional Status”, “Dietary Interventions”, “Micronutrient Deficiencies”, “Diet Therapy”, “Nutritional Assessment”, “Malnutrition”, “Sarcopenia”, “Obesity”, “Mediterranean Diet”, “Western Diet”, “Ketogenic Diet”, “Intermittent Fasting”. Boolean operators (AND, OR) were used to effectively combine these terms.

### Inclusion and exclusion criteria

The studies were selected based on the following inclusion criteria: (1) They were published within the last 20 years to ensure the relevance and timeliness of the data. (2) They included primary literature such as retrospective and prospective studies, systematic reviews, and case series.3) They focused on the association between diet, nutrition, or sarcopenia and NENs.4) They were available in English.

Exclusion criteria were: (1) studies published more than 20 years ago; (2) articles that were not available in English; (3) Studies that did not directly address the relationship between nutrition, diet, malnutrition, sarcopenia, obesity, and NENs.

### Data extraction and analysis

Two independent reviewers (S.M. and A.Z.) screened the titles and abstracts of all identified studies for eligibility. Full-text articles of potentially relevant studies were retrieved and assessed for inclusion. Discrepancies were resolved through discussion and consensus, or by consulting a third reviewer (F.P.).

Data extracted from the selected articles included study design (e.g., case report, cohort study, retrospective study), sample size, characteristics of the neuroendocrine tumors (primary site, functionality, differentiation grade), nutritional interventions, outcome measures related to the development, progression or quality of life of NEN, and conclusions regarding the impact of nutrition on NENs.

The quality of the included studies was assessed using standardized tools appropriate for each study design, such as the Newcastle-Ottawa Scale for cohort and case-control studies [22] and the Joanna Briggs Institute checklist for case reports and case series [23].

This approach aims to synthesize current knowledge on this topic, identify gaps in the literature, and suggest future research directions for the integration of nutritional strategies into the management and treatment protocols of NEN.

## Results

## Spectrum of malnutrition in NENs

### Protein-caloric malnutrition and NENs

Malnutrition is a critical factor that strongly influences the management, clinical course, and prognosis of patients with NENs. Indeed, nutritional status not only affects the quality of life but also may have a significant influence on the clinical outcomes of this patient population.

Numerous research findings underline the high prevalence of malnutrition in NEN patients [15, 18, 24–26]. However, nutritional assessments (based on weight loss, BMI, screening tools, or food intake) vary widely across studies, making an overall estimate of malnutrition in NENs difficult to obtain.

Qureshi et al. found that 14% of outpatients with GEP-NEN were at risk of malnutrition according to the Malnutrition Universal Screening Tool (MUST), demonstrating an association between nutritional risk and treatment with somatostatin analogs (SSA) [27]. Similarly, Maasberg et al. reported that 25% of patients with NEN were malnourished according to the Subjective Global Assessment (SGA) and Nutritional Risk Screening (NRS), with a further 21.7% identified as “high risk for malnutrition” [17]. Borre et al. found that 38% of NEN patients were at nutritional risk according to NRS scores and 25% had low handgrip strength, indicating reduced muscle function, known as sarcopenia [28]. In the TELECAST study, a prospective study of telotristat ethyl in refractory CS, 58% of patients with NEN and CS had metabolic and nutritional disorders [29]. In a pivotal study by Clement et al., 75% of GEP-NEN patients receiving SSA therapy were found to be malnourished based on the Global Leadership Initiative on Malnutrition (GLIM) criteria. Specifically, it was present based on low BMI in 22% of patients, based on weight loss in 30%, and based on sarcopenia in 70% of patients. Vitamin deficiencies were common, with vitamin D deficiency being present in 54% of patients [18]. More recently, Romano et al. reported a high prevalence of malnutrition in patients with advanced gastrointestinal well-differentiated NENs, with 62% of the patients being malnourished, as assessed by GLIM criteria [30].

Malnutrition in NENs has several possible causes (Table 2). NENs, especially those originating in the gastro-entero-pancreatic tract, pose unique challenges to nutritional status. Tumors at these sites can lead to malabsorption syndromes that further increase the risk of both macro and micronutrient deficiencies. Surgical procedures and hormonal releases associated with GEP-NENs exacerbate these risks, making effective nutritional management a cornerstone of comprehensive patient care [31].

**Table 2:** Causes of malnutrition and sarcopenia in gastro-entero-pancreatic neuroendocrine neoplasms (GEP-NENs)

Cause	Mechanism Impacting Nutritional Status
Direct effect of the tumor	Tumors can impair absorption of nutrients leading to macro- and micronutrient deficiencies.
Surgical Procedures	Surgeries for GEP-NENs may disrupt normal digestive functioning, exacerbating malabsorption (mainly intestinal surgery) or maldigestion (mainly pancreatic surgery).
Hormonal Releases	Hormonal disturbances due to NENs can impact metabolic processes and nutrient utilization.
Specific Functioning NEN Syndromes	Syndromes like Carcinoid Syndrome (CS), due to the release of serotonin, Zollinger-Ellison syndrome (due to gastrin release), Verner-Morrison syndrome (vasoactive intestinal peptide (VIP) release), and somatostatinoma cause severe nutritional impairments due to diarrhea and increased nutrient losses. Glucagonoma also leads to systemic effects including severe malnutrition, diabetes mellitus, weight loss, depression, necrolytic migratory erythema (a type of dermatitis), deep vein thrombosis, and anemia.
Changes in the Ghrelin System	Alterations in the ghrelin hormone due to NENs are linked to changes in appetite and metabolism, affecting nutritional status.
Therapeutic Side Effects	Treatments like somatostatin analogs (octreotide and lanreotide) may induce exocrine pancreatic insufficiency, contributing to malnutrition in up to 20% of cases. Regular monitoring and tests for fecal elastase-1 are recommended to manage these effects.

In addition, functioning NENs often cause syndromes, such as CS, Zollinger-Ellison syndrome, Werner-Morrison syndrome, and somatostatinoma syndrome, which severely impair nutritional status due to diarrheic symptoms and increased nutrient losses [5]. Glucagonoma, a rare pancreatic tumor, produces excessive glucagon leading to a unique syndrome characterized by diabetes mellitus, weight loss, necrolytic migratory erythema, anemia, and malnutrition, among other symptoms. Malnutrition and weight loss are common in patients with glucagonoma due to the tumor’s systemic effects [5]. Patients may exhibit coarse folds of the jejunum and ileum with symptoms related to malabsorption and nutrient deficiencies, with low plasma amino acid levels with a severe picture of malnutrition [32].

Additionally, changes in the ghrelin system in patients affected by NEN have been associated with their nutritional status, highlighting the complex interplay between tumor biology and nutritional status [31].

Paradoxically, malnutrition in patients with NEN is sometimes related to the therapy used to treat the disease. In particular, somatostatin analogs (both octreotide and lanreotide), which are commonly used in the treatment of functional neoplasms and non-functional G1 and G2 neoplasms as an antiproliferative treatment, are known to be associated with the occurrence of exocrine pancreatic insufficiency in up to 20% of cases [33, 34]. For this reason, it is recommended to monitor the nutritional status of patients undergoing treatment with analogs and to test for fecal elastase-1. This can help in the early detection and correction of any signs of malnutrition associated with exocrine pancreatic insufficiency.

Several studies indicate that the presence of malnutrition correlates with significantly poorer overall survival, emphasizing the importance of addressing these conditions in the therapeutic strategy [18, 35, 36]. Poor nutritional status has been reported to affect outcomes in patients receiving transcatheter arterial chemoembolization [35] and specifically in patients with pancreatic NENs [36].

### Sarcopenia and NENs

More recently, not only protein-caloric malnutrition but also sarcopenia has been documented in these patients [18–20, 31]. Specifically, Romano et al. reported a high prevalence of sarcopenia in patients with advanced gastrointestinal well-differentiated NENs, with 66.7% of the patients presenting sarcopenia, as assessed by skeletal muscle index (SMI) measured by computed tomography [30]. ​More importantly, sarcopenia resulted in significantly poorer overall survival [18, 31]. Sarcopenia is increasingly recognized as having an adverse impact on patient outcomes, including increased chemotherapy toxicity, surgical complications, and lower survival rates [19, 31, 37, 38]. In addition, CT-defined sarcopenia and myosteatosis have been reported to be prevalent in patients treated with peptide receptor radionuclide therapy (PRRT) for NENs, with sarcopenia observed in 67% of patients and myosteatosis in 71% [38]; in this study, neither sarcopenia nor myosteatosis was significantly associated with worsened survival outcomes, but 45% of patients gained weight during PRRT.

Again, the different studies used different methods to analyze sarcopenia, included heterogeneous patient populations, and were conducted at different time points after NEN diagnosis [20]. Therefore, the prevalence of sarcopenia in patients with NEN varied among studies, partly due to methodological differences and the different stages of disease at which sarcopenia was assessed.

### Obesity and NEN: The other side of the coin

Obesity plays a complex role in the context of NENs, influencing both the risk of developing these tumors and their progression, thus complicating the management of NEN patients. Obesity and its relationship with NENs have been explored in several dimensions, including the potential risks, effects, and underlying mechanisms associated. Indeed, the relationship between obesity and cancer is complex and mediated by several mechanisms, including altered hormone levels, insulin resistance, and chronic inflammation [39].

First, visceral obesity and metabolic syndrome (MetS) have been described in association with well-differentiated GEP-NENs, suggesting a link between metabolic disorders and the risk of developing GEP-NENs [40]. Moreover, obesity is associated with changes in hormone levels, such as insulin, leptin, and adiponectin, which may create a favorable environment for neoplastic cell growth. In particular, elevated insulin levels can accelerate tumor growth via the insulin-like growth factor (IGF) axis. Again, obesity is considered a state of chronic, low-grade inflammation [39]. Adipose tissue, particularly when present in excess, becomes a source of pro-inflammatory cytokines that contribute to systemic inflammation [41]. The low-grade inflammation that characterizes obesity plays a role in the development of insulin resistance, type 2 diabetes, cardiovascular disease, liver disease, and cancer [42].

Finally, obesity can complicate the management of NEN as it affects the pharmacokinetics of drugs and alters their absorption, distribution, metabolism, and excretion thereby affecting their efficacy and toxicity. However, obesity is both a risk factor for patients with NENs and also a factor associated with a better prognosis in some cancer patients, suggesting an “obesity paradox” [43]. This complex relationship underscores the need for further research to optimize patient care and outcomes [43]. On the other hand, the coexistence of sarcopenia and obesity, often referred to as sarcopenic obesity, is increasingly recognized in cancer patients [44], including those with NENs [38]. Sarcopenic obesity can lead to poorer treatment outcomes, with the combination of muscle wasting and high-fat mass presenting a particular challenge for treatment planning and prognosis.

### Micronutrients and vitamin deficiencies

Micronutrients and vitamin deficiencies, particularly in fat-soluble vitamins such as vitamin D, are a recognized challenge in the treatment of patients with NENs, particularly gastro-entero-pancreatic (GEP) NENs [24].

A pivotal study by Massironi et al. [45] involving 138 patients, found a high prevalence (68%) of 25-OH-vitamin D (25-OHvitD) deficiency, with an inverse correlation between 25-OHvitD levels and OS (*p* = 0.03, rs = -0.18) and PFS (*p* = 0.01, rs = -0.22), although this relationship was not significant in Cox proportional hazards regression. 25-OHvitD supplementation could potentially play an important role in correcting 25-OHvitD levels and influencing clinical outcomes. Robbins et al. [46] confirmed a high percentage of 25-OHvitD deficiency in NENs patients, with 31.3% of patients having 25-OH-vitD levels between 25 and 50 nmol/L, and 35.5% with < 25 nmol/L. Previous abdominal surgery, but not treatment with somatostatin analogs, predicted low 25-OH-vit-D levels. Vitamin D deficiency was also common among NEN in the study by Albertelli et al. and was associated with higher ki67 and disease progression [47]. These studies emphasize the importance of regular screening of vitamin D levels in NEN patients and suggest that targeted supplementation strategies may play a critical role in mitigating the negative effects of this deficiency.

Niacin deficiency is a significant concern in patients with CS, primarily due to the diversion of tryptophan metabolism to serotonin production. Shah et al. [48] investigated this phenomenon and discovered a significantly higher prevalence of biochemical niacin deficiency in CS patients compared to control subjects, suggesting that increased diversion of tryptophan in favor of serotonin production could result in several degrees of niacin deficiency in these patients [48]. This deficiency may contribute to the complex nutritional challenges faced by these patients and emphasizes the need for comprehensive nutritional assessments and interventions in the treatment of CS.

Emerging research, including a study by Walter et al. [49], highlights the intricate relationship between folic acid metabolism, DNA repair phenotypes, and neuroendocrine lung tumors. Alterations in folic acid metabolic pathways and DNA repair mechanisms may account for the heterogeneity and differential response to therapy observed in neuroendocrine lung cancer subtypes, including typical and atypical carcinoids, large-cell neuroendocrine carcinomas (LCNEC) and small cell lung carcinomas (SCLC) [49]. Further research in this area may lead to improved strategies for the treatment of NENs, including personalized approaches based on specific molecular profiles associated with folic acid signaling pathways.

The role of vitamin B12 in oncology is a subject of ongoing debate. Vitamin B12 receptors have been identified in various tumors, and there are studies suggesting that a relative deficiency of vitamin B12 may slow the growth of certain tumors [50]. On the other hand, the presence of vitamin B12 receptors on tumor cells offers the possibility of targeting this vitamin for the administration of certain therapies [51]. This suggests that vitamin B12 plays a complex role in cancer biology and may influence tumor growth dynamics. Unfortunately, there is a lack of research on the role of vitamin B12 in NEN.

Similarly, the specific benefits of vitamin C in preventing or treating NENs have not yet been sufficiently researched, despite its recognized antioxidant properties. Currently, there is no comprehensive evidence on how vitamin C supplementation might affect the outcomes of NEN patients.

Overall, vitamins deficiencies highlight the complex interplay between tumor activity, nutrient absorption, and the body’s nutritional reserves, underscoring the need for targeted nutritional support in these individuals.

## Nutritional assessment in NENs

The cornerstone of effective NENs management involves a thorough nutritional assessment to identify specific needs and potential deficiencies [15].

Accurate assessment of nutritional status in NEN patients must consider not only traditional markers like BMI but also detailed evaluations of body composition. Techniques such as bioelectrical impedance analysis (BIA), dual-energy X-ray absorptiometry (DEXA), computed tomography (CT) scans, or even ultrasound [52] provide valuable insights into muscle mass, fat distribution, and sarcopenia, which are critical factors in the prognosis and management of NENs [38]. For instance, sarcopenic obesity—a condition characterized by the presence of both excess fat and muscle loss—presents a unique challenge in NEN management, as it is associated with worse outcomes [38]. Regular monitoring and assessment of body composition should be integrated into the nutritional management plans for NEN patients to better tailor interventions and improve clinical outcomes.

Tools such as the SGA, NRS, and GLIM criteria serve as essential tools for this purpose, even if they are far from being standardized [26, 53–55]. This assessment is crucial for adapting nutritional interventions that can improve patient’s quality of life and treatment outcomes [20].

Regular screening not only for malnutrition but also for protein intake and vitamin deficiencies should be an integral part of every GEP-NEN patient’s care [24]. Personalized nutritional strategies are crucial in addressing the unique nutritional challenges of NENs patients.

## Personalized nutritional strategies

### Impact of diet on NEN

The relationship between dietary habits and the risk or progression of NENs is an area of growing interest. Research into the relationship between dietary patterns and the risk or progression of NENs shows that both macro- and micronutrients may play an important role and that certain dietary patterns may directly or indirectly influence tumor growth by modulating the body’s hormonal balance and inflammatory processes. For instance, a high-fat Western diet, characterized by a high intake of red meat, processed foods, and saturated fats, has been associated with increased growth of certain specific types of NEN [56], particularly pancreatic NENs. This suggests that dietary fats may influence tumor proliferation via metabolic pathways.

Conversely, the MD, known for its richness in fruits, vegetables, whole grains, fish, and olive oil, is associated with lower insulin levels and improved immune responses, which may also provide a protective mechanism against the development of NEN. The protective effect is attributed to the reduction of oxidative and inflammatory cell processes, DNA damage, cell proliferation, survival, angiogenesis, inflammation, and metastasis [15, 16, 57]. Beyond its antioxidant and anti-inflammatory nutrient content, MD may help reduce the incidence of cancer, including NEN, by providing specific polyamines whose role in disease prevention has recently been reported. Spermidine, a natural autophagy-promoting polyamine, extends the lifespan of all animal species in an autophagy-dependent manner [58]. By promoting autophagy and contrasting mitochondrial dysfunction, a diet enriched in spermidine could be considered to prevent NEN [59], and potentially increase the efficacy of cancer therapies [60]. A pivotal study examining the nutritional status and adherence to the MD in patients with gastro-entero-pancreatic NENs (GEP-NENs) found that deviations from the MD, specifically lower intake of plant protein and complex carbohydrates, were associated with more aggressive tumor features [25]. These findings emphasize the potential of dietary interventions to modulate disease progression [15]. Despite these promising findings, larger, longitudinal studies must be conducted to confirm these associations and fully understand the impact of diet on NENs.

The therapeutic potential of the ketogenic diet (KD) and intermittent fasting (IF) in the treatment of various cancers, including NENs, is also being investigated [15, 61]. The KD, characterized by a high-fat, low-carbohydrate composition with an adequate amount of high-protein, is emerging as a promising approach for the treatment of various cancers, including gynecological and neurological cancers [62]. By inducing a state of ketosis, in which the body uses fats instead of carbohydrates for energy, the KD may slow the growth of certain cancer cells by depriving them of glucose, a crucial nutrient for their proliferation. This mechanism takes advantage of the altered metabolism of cancer cells and targets tumor growth by modulating gene expression and the tumor microenvironment [63–65]. Specifically, in the context of NENs, one hypothesis is that the KD could improve insulin resistance, which could be beneficial not only in some forms of secreting NETs associated with hyperinsulinemia but also in regulating neoplastic growth. Indeed, insulin signaling plays a crucial role in cancer regulation and influences tumorigenesis, tumor progression, and response to treatment. Moreover, the PI3K-Akt-mTOR signaling pathway plays an important role in islet cell proliferation and is hyperactivated in human non-functioning pancreatic NENs. One study has shown that KD leads to lower PI3K-Akt-mTOR signaling in islet cells and significantly reduces the formation and progression of pancreatic NENs [66].

However, despite these potential benefits of KD, this diet should be used with caution due to potential symptoms and effects related to fat malabsorption.

As regards IF, which includes strategies such as one or two non-consecutive days per week of 24-hour fasting, there are currently no studies that have been conducted specifically in NEN patients, although preclinical studies have shown promising effects on cancer [15]. It is speculated that the anti-inflammatory effects and weight loss associated with IF could improve tumor response to cancer treatments in NEN patients. Moreover, the role of chronobiology and circadian rhythms in cancer management has gained significant attention in recent years. Dietary patterns such as intermittent fasting may influence these rhythms, potentially improving metabolic regulation and enhancing the effectiveness of therapeutic interventions [67–69]. However, as with KD, a possible anti-Warburg effect may also occur with IF. As there is no direct evidence in NEN patients, further research is required before recommendations can be made.

Finally, it has been reported that certain diets could potentially be considered as adjuvant disease modulators along with NENs treatment and could alleviate symptoms associated with both NEN syndromes and NENs treatments. For example, MD may help manage gastrointestinal side effects associated with NEN treatments [70]. KD has been suggested to increase the efficacy of pharmacological treatments by acting on certain signaling pathways (IL-6, VEGF, PI3K/AKT/mTOR) [61]. It has been suggested to potentially reduce tumor growth and chemotherapy-related toxicity, especially diarrhea. On the other hand, as with any therapeutic intervention, the potential side effects must be considered. Indeed, the balance between the benefits of IF and the harms of inadequate caloric intake still needs to be better understood [71], which could lead to changes in nutritional status such as malnutrition and sarcopenia, and have a negative impact on patient outcomes. In addition, these diets could exacerbate certain deficiencies, including niacin deficiency in carcinoid syndrome (CS) and vitamin D deficiency, commonly observed in patients with NENs.

### Role of Food and specific nutrients in NENs

Investigating specific nutrients’ roles, the influence of dietary patterns on tumor biology, and the individual variability in response to dietary interventions offer invaluable insights, into where diet becomes a pivotal component of personalized cancer care strategies. Current research highlights significant gaps in our understanding of the specific dietary needs and interventions beneficial for NEN patient outcomes, even if the most equilibrated diet seems to be the MD, which assures a balanced caloric intake, and beneficial effects on both nutritional status and disease outcome [16, 57, 72].

An analysis of 26 GEP-NET patients revealed significant nutritional bad habits, including high-fat consumption, insufficient intake of vitamins D, E, folates, niacin, calcium, magnesium, iodine, and potassium, along with an excess of sodium and cholesterol in these patients [73]. As already mentioned for MD, vegetables, fruits, nuts, fish, and olive oil, and low consumption of red meat, has been associated with lower risks of various cancers [57], including NENs [72]. These nutritional considerations highlight the need for a well-balanced diet to support patient health, even if no specific foods or nutrients can alter the disease course.

### Individualized nutritional approach in NENs

Nutrition plays a significant role in the management of NENs. Malnutrition and poor dietary habits potentially negatively affect the prognosis of patients with NENs, therefore proper nutritional status is crucial for managing complications and improving outcomes [53].

In addition to ensuring the need for calories and proteins, the correction of vitamin and micronutrient deficiencies is also very important. For instance, vitamin D not only plays a role in bone health but also has an impact on tumor growth and patient outcomes [45]. Not only vitamin D but also MART-10, a 1α,25-dihydroxyvitamin D3 analog, has been shown to inhibit the growth of neuroendocrine tumor cells by inducing G0/G1 cell cycle arrest and apoptosis [74], suggesting that vitamin D and its analogs might play a role in NEN treatment strategies by inhibiting tumor growth and inducing cell death.

As mentioned above, niacin deficiency is common in CS, and some foods can exacerbate CS. For this reason, patients with CS need some special attention. This involves avoiding foods and drinks that may trigger or worsen these symptoms, such as alcohol, especially red wine, and beer, fermented foods, which can be high in tyramine (e.g., matured cheeses, smoked fish, cured meats), large meals, which may trigger symptoms due to the volume of food, and spicy foods [70]. This underscores the need for dietary modification as part of comprehensive and personalized patient management [75].

This tailored approach to nutrition also extends to other particular conditions. Indeed, a critical aspect of developing a nutritional plan includes assessing whether the patient has undergone surgery. For instance, pancreatic surgery, such as a duodenal-pancreatectomy or total pancreatectomy, requires pancreatic enzyme replacement therapy to prevent maldigestion and worsen diarrhea, which could already be a symptom of the NEN itself. Moreover, patients who have undergone a cholecystectomy may experience post-cholecystectomy diarrhea, leading to nutritional deficiencies. Additionally, in cases involving intestinal resections, it’s vital to assess whether the terminal ileum is affected; in such scenarios, vitamin B12 supplementation is necessary (Table 3).

**Table 3:** Nutritional assessment and personalized dietary strategies

	Tools/Strategies	Purpose/Impact
Nutritional Assessment	Subjective Global Assessment (SGA), Nutritional Risk Screening (NRS), Global Leadership Initiative on Malnutrition (GLIM) criteria, ESPEN guidelines Bioelectrical impedance analysis (BIA), dual-energy X-ray absorptiometry (DEXA), computed tomography (CT) scans, muscle ultrasound to evaluate body composition	Identifies specific nutritional needs and deficiencies to tailor nutritional interventions; crucial for improving quality of life and treatment outcomes
Nutritional Support	Attention to protein intake, management of nutrient deficiencies	Mitigates disease-related malnutrition and sarcopenia; critical for patient care
Screening for Vitamin Deficiencies	Regular screening for vitamin deficiencies, especially vitamin D deficiency. Correction of micronutrient deficiencies (e.g., Vitamin D, niacin)	Integral part of care for GEP-NEN patients to develop effective management strategies
Personalized Nutritional Strategies	Carcinoid syndrome (CS): nutritional support and correction of niacin Zollinger Ellison syndrome: nutritional support, correction of micronutrient deficiencies due to acid-related disease (iron, folic acid) Glucagonoma syndrome: correction of severe protein-caloric malnutrition, attention to diabetes usually coexisting Somatostatinoma syndrome: nutritional support, attention to diabetes usually coexisting, and fat-soluble vitamins due to maldigestion Non-functioning tumors: Consider novel dietary approaches (IF, KD) Patient undergone surgery: in pancreatic resection give pancreatic enzyme replacement therapy (PERT); in distal ileal resection replace vitamin B12, if distal ileum is resected.	Addresses unique nutritional challenges, preserves muscle mass, prevents sarcopenia, and potentially inhibits tumor growth.

Future research should focus on clarifying the effectiveness of specific nutritional approaches. Novel dietary approaches such as IF and KD are being investigated, but their application in the treatment of NEN requires further research to conclusively demonstrate their efficacy and safety.

It should be acknowledged that the prevention and treatment of cancer-related malnutrition has not consistently shown clinical benefit in terms of improving nutritional status or prolonging survival. One of the possible reasons is related to the increased inflammatory response associated with the presence of a tumor or with the administration of cancer therapies. Bargetzi L et al. have shown that early and individualized nutritional support improves 30-day survival in hospitalized cancer patients at risk of malnutrition [76]. However, the clinical benefit disappears when patients are stratified according to the severity of the inflammatory response, which is roughly assessed by circulating levels of C-reactive protein [77]. In line with these observations, modulation of the inflammatory response could be an important target to optimize the metabolic and anabolic benefits of nutritional supplementation. Omega-3 fatty acids have been shown to modulate the inflammatory response by increasing the production of less active inflammatory mediators compared to those from omega-6 fatty acids. In a systematic review with meta-analysis, de van der Schueren et al. showed that standard oral supplements during chemo-radiotherapy did not prevent weight loss, while omega-3-enriched oral supplements lead to improved body weight [78], thus contrasting malnutrition and the inflammatory milieu that favors the neoplastic microenvironment.

In the presence of sarcopenia, consuming high-quality protein from sources like fish, eggs, and legumes can significantly help in maintaining muscle mass and preventing sarcopenia. Fish contains essential nutrients such as omega-3 fatty acids, proteins, vitamin D, magnesium, and carnitine, which positively affect muscle metabolism and help maintain muscle performance [79]. However, it has been suggested that muscle mass may be resistant to supplementation due to the presence of senescent cells, that are no longer able to proliferate but remain in the tissue and secrete a specific set of molecules called senescent-associated secretory phenotypes (SASP) [80]. In muscles, SASP reduces the ability to recover from trauma [81], and it could be speculated that they are also involved in resistance to the anabolic effects of supplementation. This hypothesis is supported by the fact that the use of senolytic drugs reverses cancer cachexia in animal models [82]. Therefore, it could be speculated that sarcopenia may be a marker of aging and cancer-related wasting may be the phenotype of accelerated ageing.

In the case of obese individuals with NENs, diets high in processed foods, sugars, and unhealthy fats contribute to obesity and may exacerbate inflammation and tumor growth [43] and may influence the progression of NENs, therefore, even if seems to have a protective role, should be avoided.

## Discussion

The current research landscape on the role of nutrition in the treatment of NENs underscores a significant knowledge gap, particularly in identifying specific nutritional needs and interventions that could positively influence patient outcomes. This gap highlights the need for more rigorous studies, including randomized controlled trials and longitudinal observational studies, to develop evidence-based nutritional guidelines tailored to NEN patients. To date, only a handful of studies and reviews have approached this crucial area [15, 75], which is surprising given the potential of nutritional intervention for the long-term survival of NEN patients. This underscores the urgent need to expand our research focus to address these critical gaps.

Incorporating patient-reported outcomes and quality of life measures into such studies could provide a more comprehensive understanding of how nutritional and dietary interventions and nutritional support affect NEN patients. This holistic approach could provide actionable insights that significantly improve patient care.

Innovative dietary interventions, including IF, hold promise for improving the treatment of NEN and the quality of life of patients. The potential of these novel dietary strategies to influence the progression of NEN should be thoroughly investigated. However, the impact of such interventions, including any adverse effects such as the risk of malnutrition or worsening of sarcopenia, must be carefully weighed to ensure they do not inadvertently compromise patient health.

Understanding the role of specific nutrients, the impact of different dietary patterns on tumor biology, and the interplay between the gut microbiota and NEN will be critical to discovering new therapeutic opportunities. Understanding individual variability in response to diet — considering genetic, metabolic, and environmental factors — is also crucial. Indeed, emerging evidence suggests that genetic and molecular differences between men and women may influence the pathophysiology, clinical manifestations, and treatment outcomes of various cancers, including NENs [83, 84]. These gender differences are largely attributed to the role of sex hormones, such as estrogen and testosterone, which can modulate gene expression in tumor cells. For instance, estrogen receptors are known to play a role in some cancer types by influencing cell proliferation, apoptosis, and metastasis [85]. Future research should focus on delineating these gender-based differences in NENs, which could pave the way for tailored therapies that consider the patient’s sex as a factor in treatment planning.

Implementing effective nutritional interventions in NEN patients presents several challenges. These include ensuring adherence to dietary recommendations, addressing different nutritional needs depending on tumor type and treatment, and fostering multidisciplinary collaboration for comprehensive care. Personalized diet plans that reflect the patient’s lifestyle, preferences, and nutritional needs are critical to success [15]. The variability of nutritional needs, influenced by tumor location and side effects of treatments, necessitates a flexible, tailored approach.

Moreover, the clinical context of NEN, characterized by their indolent behavior and long-term survival, underlines the need for targeted nutritional support. Such support should aim to correct deficiencies and improve overall nutritional status to mitigate the negative effects of malnutrition and sarcopenia [26, 53].

The management of NENs is complex and requires a multidisciplinary approach to address the multifaceted needs of patients. Central to this team is the involvement of dietitians or qualified nutritionists who play a critical role in assessing and managing the nutritional needs of NEN patients. Recent studies highlight the significant impact of tailored nutritional strategies on patient outcomes [72]. Dietitians and nutritionists are essential for developing individualized care plans that address malnutrition, micronutrient deficiencies, and other diet-related issues that are common in NEN patients. Their expertise ensures that nutritional interventions are not only effective but also safe, considering the complex interactions between diet, NEN pathophysiology, and treatment modalities. A strong collaboration among oncologists, endocrinologists, surgeons, gastroenterologists, and nutrition experts is therefore crucial in optimizing the care and improving the quality of life for NEN patients.

**Fig. 1:**
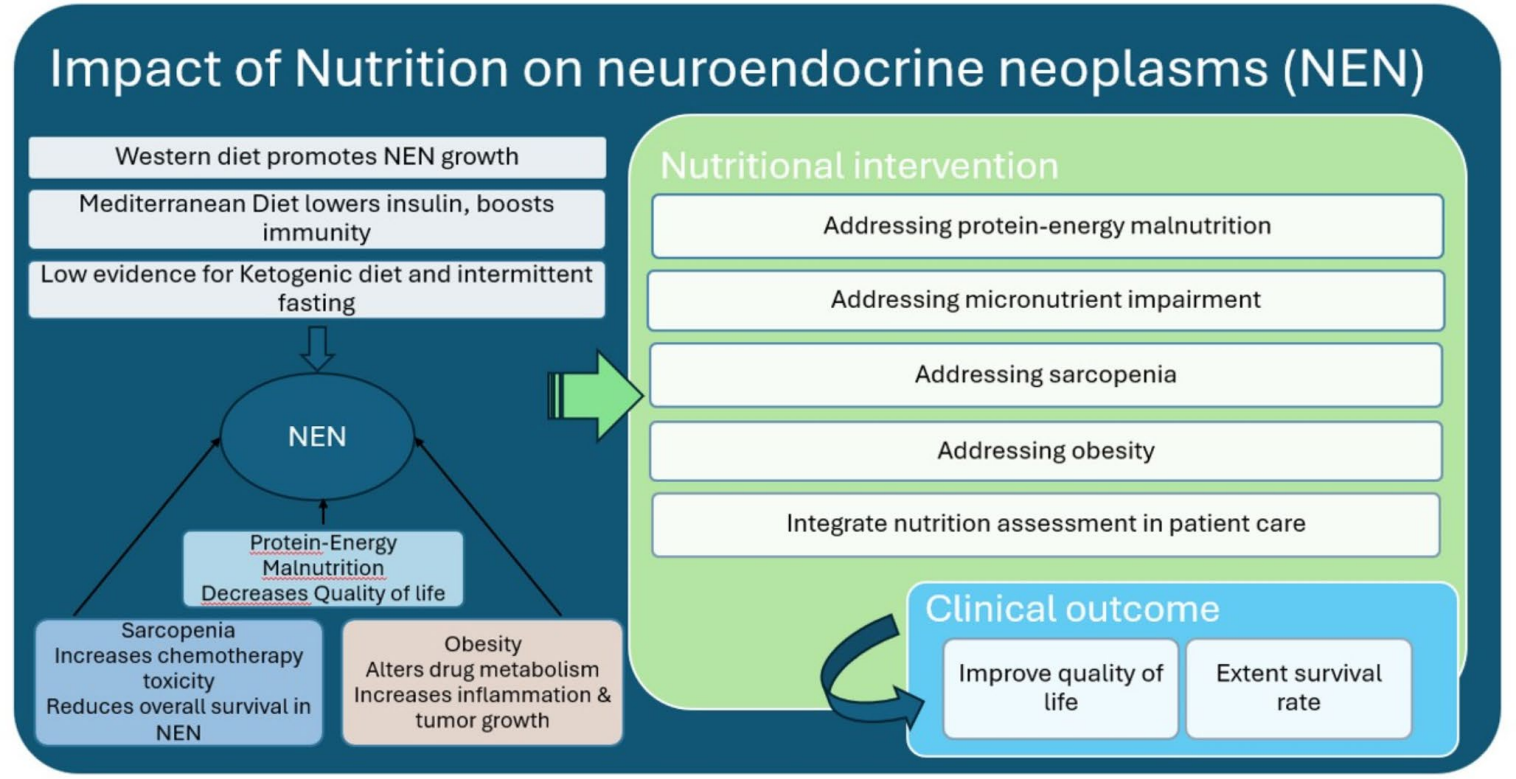
Bidirectional Impact of Nutrition in Neuroendocrine Neoplasms (NENs): This figure illustrates the dual influence of dietary choices and nutritional interventions on the progression and management of NENs, with a particular emphasis on the critical role of targeted nutritional strategies in addressing malnutrition, sarcopenia, and obesity. It also connects these nutritional interventions to potential clinical outcomes, such as improved quality of life and extended survival rates

While further research is needed to unravel the complex interactions between diet and NENs, the available evidence underscores the untapped potential of nutritional interventions to enrich a comprehensive approach to the management of NEN (Fig. 1). Addressing this need will not only fill the existing knowledge gaps but will also significantly improve the quality of care for patients with NENs.

## Conclusion

The complex interplay between nutrition, sarcopenia, and NENs highlights the need for multidisciplinary management strategies that incorporate nutritional support and interventions aimed at preventing or mitigating malnutrition and sarcopenia. Further research is essential to unravel the detailed mechanisms underlying these relationships and to develop optimized therapeutic approaches that improve the quality of life and survival rates of NEN patients.

## Electronic supplementary material

Below is the link to the electronic supplementary material.

Supplementary Material 1

Supplementary Material 2
