# Supplementary material for: Nutritional aspects in neuroendocrine neoplasms. bridging the gap between dietary interventions and cancer care strategies: a scoping review
Source: J Endocrinol Invest. 2024 Oct 12;48(2):269–81. doi: 10.1007/s40618-024-02462-8 (PMC11785655; doi:10.1007/s40618-024-02462-8)
Supplement: Supplementary file 1 — Supplementary Material 1 [file 40618_2024_2462_MOESM1_ESM.docx]

**Editor-in-Chief**                                             
Uberto Pagotto, MD, PhD
University of Bologna 
Department of Medical and Surgical Sciences
Bologna, Italy

Thursday, July 11, 2024

Dear Editor-in-Chief,

I am writing to submit our manuscript entitled "Nutritional Aspects in Neuroendocrine Neoplasms: Bridging the Gap Between Dietary Interventions and Cancer Care Strategies: A Scoping Review" for consideration for publication in the [*Journal of Endocrinological Investigation*](https://link-springer-com.unimib.idm.oclc.org/journal/40618). Our study highlights the critical role of nutritional management in the comprehensive treatment of patients with neuroendocrine neoplasms (NENs) and identifies significant research gaps that could enhance patient care and outcomes.

Our review summarizes the findings from an extensive literature search focusing on the interactions between nutritional assessments, dietary interventions, micronutrient deficiencies and their impact on the progression and outcomes of patients with NEN. The results highlight the lack of targeted research in this area and emphasize the potential of personalized nutritional strategies to improve clinical outcomes.

This manuscript has not been published elsewhere and is not under consideration by any other journal. Given the journal’s commitment to advancing knowledge of nutritional interventions in disease, we believe that our review provides valuable insights that are in line with the scope of [*Journal of Endocrinological Investigation*](https://link-springer-com.unimib.idm.oclc.org/journal/40618), offering a comprehensive overview of how nutrition can play a central role in care strategies for NEN.

We have adhered to all submission guidelines outlined in your Instructions for Authors.

Thank you for considering our work for publication in [*Journal of Endocrinological Investigation*](https://link-springer-com.unimib.idm.oclc.org/journal/40618). We look forward to the opportunity to contribute to the journal and would be happy to discuss further.

Sincerely,

Sara Massironi, MD, PhD

Division of Gastroenterology, San Gerardo Hospital, Via Pergolesi 3, Monza, Italy

Tel. +39-039-2332317; Fax: +39-039-2300129;

E-mail: [sara.massironi@libero.it](mailto:sara.massironi@libero.it)
