# Supplementary material for: Nutritional aspects in neuroendocrine neoplasms. bridging the gap between dietary interventions and cancer care strategies: a scoping review
Source: J Endocrinol Invest. 2024 Oct 12;48(2):269–81. doi: 10.1007/s40618-024-02462-8 (PMC11785655; doi:10.1007/s40618-024-02462-8)
Supplement: Supplementary file 2 — Supplementary Material 2 [file 40618_2024_2462_MOESM2_ESM.docx]

Uberto Pagotto, MD, Professor
Editor in Chief
Journal of Endocrinological Investigation

August 22nd, 2024

Dear Editor,

Thank you for considering our manuscript, "Nutritional Aspects in Neuroendocrine Neoplasms. Bridging the Gap Between Dietary Interventions and Cancer Care Strategies: A Scoping Review," for potential publication in the Journal of Endocrinological Investigation.

The authors wish to thank the Editors and the reviewers for their insightful comments and corrections, which have helped to improve the quality of our work.

We have thoroughly addressed all the comments and suggestions provided by the reviewer and we hope that the revisions and clarifications meet the reviewer's expectations and improve the manuscript's clarity and rigor.

All changes are tracked in the revised manuscript.

Below is a detailed point-by-point response to the reviewer’s comments:

Reviewer #1: The authors provide a comprehensive and interesting review on the nutritional aspects of NEN management, a clinical setting where research is still limited. Aim of this review is to summarize the current state of knowledge, highlight research gaps, and underscore the significance of nutrition in the comprehensive care of NEN patients. Authors report significant gaps exist in current research, particularly in understanding the specific nutritional needs of NEN patients and how tailored nutritional interventions can improve clinical outcomes. This issue is particularly interesting as nutritional treatment of NENs have been in a state of neglect for a long time.
**We thank the reviewer for his appreciation.**
My two main observations are:
1. Introduction: the concept of sarcopenia and obesity should be introduced and the types of NEN should be better detailed;

**We have introduced the concepts of sarcopenia and obesity (page 4, lines 81-94).**

**We have detailed the classification of NENs into well-differentiated neuroendocrine tumors (NETs) and poorly differentiated neuroendocrine carcinoma (NECs) (page 3, lines 51-55).**

2. Results: these should be better presented to improve understanding. In particular, according to this reviewer, three main chapters should be presented: 1) NEN, 2) NEN and sarcopenia and 3) NEN and obesity. In each, the following three subchapters should be detailed (nutrition, foods and dietary therapies).

**Thank you for your valuable feedback. We organized the results around the key nutritional challenges—malnutrition, sarcopenia, obesity, and micronutrient deficiencies—to highlight their interconnectedness in NENs. This approach allows us to illustrate how these conditions impact each other and NEN management. We also included detailed discussions on relevant nutritional strategies, foods, and dietary therapies in a specific section (see “3. Personalized nutritional strategies”). Additionally, we provided a comprehensive overview of Nutritional Assessment and personalized nutritional strategies, ensuring a holistic and clinically relevant discussion. These revisions were made to align with the suggestions of Reviewer 2 as well. (See Results)**

Introduction
- NENs should be better classified as well-differentiated neuroendocrine tumours (NETs) and poorly differentiated neuroendocrine carcinoma (NECs). Additionally, metabolic and nutritional complications should be addressed.

**We have detailed the classification of NENs into well-differentiated neuroendocrine tumors (NETs) and poorly differentiated neuroendocrine carcinoma (NECs) (page 3, lines 51-55). Additionally, we have addressed metabolic and nutritional complications (page 4, lines 81-94).**
- The authors report: "This review aims to provide a comprehensive examination of the current literature on the prevalence, causes and clinical impact of malnutrition and sarcopenia in NENs, identify existing knowledge gaps, and suggest future research directions to improve the management and outcomes of patients with NENs". However, in the introduction the authors do not address the issue of malnutrition in NEN patients. Information on the importance of malnutrition in NEN should be introduced in detail. In particular the multiple associations between sarcopenia - nutrition and NEN.

**We expanded the introduction to discuss the significant impact of malnutrition on NEN patients and highlight the prevalence and implications of malnutrition in NEN patients, illustrating its role in worsening sarcopenia and affecting overall clinical outcomes. We also have emphasized the multifaceted relationship between malnutrition, sarcopenia, and NENs, underscoring the critical need to address these issues in comprehensive care strategies.**

**Furthermore, we introduced the role of obesity and visceral adipose tissue in NEN patients and the associations between inflammation - nutrition and NEN should also be reported.**

**The possible role of obesity and visceral adipose tissue in NEN patients, including the associations between inflammation, nutrition, and NEN has been expanded (page 4, lines 81-94).**

Methods
- How many articles were included? What kind of articles were they? More details should be provided on this point.
- A flowchart is needed to better explain the inclusion and exclusion criteria.

Data Extraction and Analysis
- A summary table would be very useful to readers.
**Thank you for your valuable feedback on our manuscript. We appreciate the opportunity to address your comments and provide further clarity on our methodology. In our revised Methods section, we now have specified that our systematic search included primary literature such as retrospective and prospective studies, systematic reviews, and case series. We have now added specific details about the number and types of articles included in our review. We indeed have included a PRISMA flow diagram in Table 1 to provide a clear visualization of the study selection process for our review. This flowchart outlines the steps taken from the initial identification of records to the final inclusion of studies, detailing the number of records at each stage and the reasons for exclusions.**
Results
This chapter should be reformulated.
1) NEN, 2) NEN and sarcopenia and 3) NEN and obesity.
In each, the following three subchapters should be detailed:
- Nutrition and NEN. The studies that have evaluated the impact of nutrients on NEN should be detailed here;
- Foods and NEN. The results of studies on specific foods and NEN (Meat, fish, eggs, vegetables, ...) should be addressed here;
- Dietary therapies and NEN. The role of different dietary patterns in NEN must be detailed here. Mediterranean diet, ketogenic diets, intermittent fasting (chronobiology and circadian rhythms?). Furthermore, dietary therapies, their indications and contraindications and the rationale for use in NEN should be better explained.
**Thank you for your valuable feedback. We organized the results around the key nutritional challenges—malnutrition, sarcopenia, obesity, and micronutrient deficiencies—to highlight their interconnectedness in NENs. This approach allows us to illustrate how these conditions impact each other and NEN management. We also included detailed discussions on relevant nutritional strategies, foods, and dietary therapies in a specific section (see “3. Personalized nutritional strategies”). Additionally, we provided a detailed exploration of various dietary patterns, including the Mediterranean diet, ketogenic diet, and intermittent fasting, emphasizing their potential roles in modulating chronobiology and circadian rhythms in NEN patients. These revisions were made to align with the suggestions of Reviewer 2 as well. (See Results)**

Prevalence of malnutrition/sarcopenia in NEN
- Lines 175 - 177: Please add specific references
- "Numerous research findings underline the high prevalence of malnutrition in NEN patients (16)." Numerous studies, just one reference?
- Here the studies must be detailed. What was the case? Type? What type of NEN did the patients have? Were they on drug therapy?
**Thank you for your comments and for pointing out the need for additional details and references in the section on the prevalence of malnutrition and sarcopenia in NEN patients. We have addressed your concerns as follows:**

**- We have included additional references to support the statement regarding the high prevalence of malnutrition in NEN patients (references 15, 18, 24-26)**

**-Numerous Studies, One Reference?: We have expanded the citation to include multiple studies that collectively underline the high prevalence of malnutrition in NEN patients (references 15, 18, 24-26).**

**-Detailing the Studies: Additionally, we have expanded the descriptions of these studies to clarify the patient populations, types of NENs, and whether patients were undergoing drug therapy, as seen in the work by Qureshi et al., Maasberg et al., Borre et al., and Romano et al. These studies reflect the diverse clinical scenarios in NEN management, including the impact of somatostatin analog therapy on nutritional status.**
Impact of malnutrition on clinical outcome
- Lines 205 - 207: Please add specific references
The other side of the coin: Obesity and NENs
- Lines 287 - 292: Please add specific references;
- The role of endocrine dysfunction associated with obesity and the role of nutrition should be addressed
**Thank you for your valuable comments. We have made the following revisions to address your concerns:**

**- Impact of Malnutrition on Clinical Outcomes: We have added specific references to support the statement regarding the impact of malnutrition on clinical outcomes in NEN patients. These references now include studies that highlight the significant correlation between poor nutritional status and adverse clinical outcomes, including reduced overall survival and increased complications during treatment (references [17, 18, 35, 36]).**

**-Obesity and NENs: We have included additional references to substantiate the discussion about the relationship between obesity and NENs. These references provide evidence of how obesity, particularly visceral obesity, and metabolic syndrome, influences the risk and progression of NENs (references [39, 40, 42, 43]). Furthermore, we have expanded the section to address the role of endocrine dysfunction associated with obesity and its implications for NENs. Specifically, we have discussed how obesity-related endocrine changes, such as insulin resistance and alterations in adipokine levels, create a pro-tumorigenic environment that can exacerbate NEN progression. Additionally, the role of nutrition in managing obesity-related risks in NEN patients has been elaborated to provide a more comprehensive overview.**

Discussion/Conclusion
A summary figure summarizing the importance of nutrition in NEN, NEN + sarcopenia and NEN + obesity would be very useful.
**Thank you for your valuable suggestion. We have modified Figure 1 to now present a comprehensive overview that highlights the importance of nutrition/malnutrition in NEN, NEN + sarcopenia, and NEN + obesity.**

**The updated Figure 1 illustrates the bidirectional impact of different dietary patterns, such as the Western and Mediterranean diets, on NEN growth. It also emphasizes the critical role of targeted nutritional interventions in addressing malnutrition, sarcopenia, and obesity. Furthermore, the figure connects these nutritional strategies to potential clinical outcomes, including improved quality of life and extended survival rates.**

Further considerations:
- Genetic and molecular differences between men and women have a role in the differing pathophysiology, clinical signs, incidence, and treatment outcome of different cancers. These gender differences can be attributed to sex hormones that in turn modulate gene expression in various tumors. This aspect might allow better tailoring of the management of these rare group of neoplasms. However, little is known of gender differences on neuroendocrine neoplasms. This concept should be analysed;

**Thank you for your insightful suggestions. We have added a section that explores the genetic and molecular differences between men and women in the context of NENs. This section discusses how sex hormones modulate gene expression and influence the pathophysiology, clinical signs, incidence, and treatment outcomes of various cancers, including the potential implications for NEN management. Although data on gender differences specific to NENs are limited, we highlight the importance of this aspect for better tailoring management strategies (page 19, lines 469-477)**.
- The importance of body composition in the correct assessment of the nutritional status of NEN patients should be addressed;

**The manuscript has been updated to include a discussion on the significance of body composition in the accurate assessment of nutritional status in NEN patients. We emphasize the role of techniques such as CT, MRI, and also US in evaluating muscle mass and fat distribution, which are critical for identifying malnutrition and sarcopenia, especially in the presence of obesity (page 12, lines 290-296).**

- The role of the multidisciplinary team in the complex management of NEN and in particular of the registered dietician or qualified nutritionist, recently addressed in Journal of Endocrinological Investigation (See PMID: 37697017), should be clearly highlighted.
**We have highlighted the critical role of the multidisciplinary team in the complex management of NENs, with particular emphasis on the importance of involving a registered dietitian or qualified nutritionist. We referenced recent literature, including PMID: 37697017, to underscore the importance of developing personalized nutritional strategies that improve patient outcomes (page 20, lines 488-498).**

Reviewer #2: In this review the Authors summarized the current state of knowledge on Nutritional Aspects in neuroendocrine neoplasms, highlight research gaps.
The paper is interesting and well written.

However, I have some comments.
The "aim" of the review should be better explained, also including the role of obesity, which is examined in the paper.

**Thank you for the suggestion. We have clarified the review's aim in the introduction, explicitly including the exploration of obesity's role in NENs, focusing on its impact on risk and disease progression through mechanisms like altered hormone levels and chronic inflammation (Page 4, lines 95-101).**

Please, define the acronyms MEN1, VHL, TSC2 and NF1 in the introduction.
**We have defined the acronyms MEN1, VHL, TSC2, and NF1 in the introduction to ensure clarity for all readers (Page 3, lines 61-63)**
Methods: please add the end of the research period.

**Thank you for pointing this out. We have added the start and end dates of the research period to the Methods section for clarity (Page 5, lines 104-106).**
Was "obesity" included among the keywords?

**Yes, "obesity" was included among the keywords to ensure comprehensive coverage of the topic (see Methods).**
I suggest including a flowchart for the inclusion and exclusion of studies
**Thank you for the suggestion. We have included a PRISMA flowchart in the Methods section to visually represent the inclusion and exclusion criteria for the studies reviewed.**
The paragraph " Etiological Factors" should be unified with the previous one "Prevalence of malnutrition/sarcopenia in NEN"
**We appreciate the reviewer's suggestion. Upon review, we have unified the paragraph "Etiological Factors" with "Prevalence of malnutrition/sarcopenia in NEN" to enhance the coherence of the manuscript.**
Figure: Please use "intermittent fasting" instead of "intermittent diet"

**We have updated the figure to use "intermittent fasting" instead of "intermittent diet."**

We sincerely appreciate the constructive feedback provided by the reviewers. We have carefully considered all comments and made the necessary revisions to enhance the clarity, comprehensiveness, and rigor of our manuscript. We believe these changes significantly strengthen our review and address the concerns raised by the reviewers. We hope the revised manuscript now meets the high standards of the Journal of Endocrinological Investigation and is suitable for publication.

Thank you for your consideration.

Sincerely,

Sara Massironi, MD PhD
